# Supplementary material for: Measurement validity of an electronic training device to assess breathing characteristics during inspiratory muscle training in patients with weaning difficulties
Source: PLoS One. 2021 Aug 26;16(8):e0255431. doi: 10.1371/journal.pone.0255431 (PMC8389486; doi:10.1371/journal.pone.0255431)
Supplement: S1 Fig — Example of the comparison of the calculation of the work of breathing performed on the raw signal of the portable spirometer (Panel A) and the calculation of the smoothed signal with a time constant of 0.13s in the Spike 2 software (Panel B). (PDF) [file pone.0255431.s001.pdf]

S1 Fig. Calculation of work of breathing by the use of unsmoothed versus smoothed pressure-volume loops

A

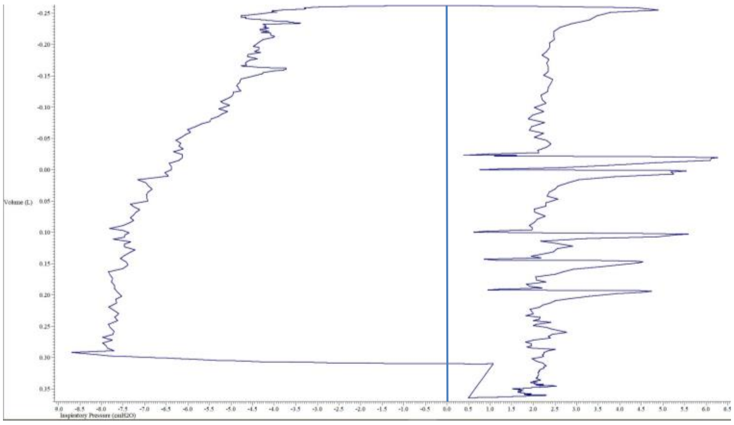

B

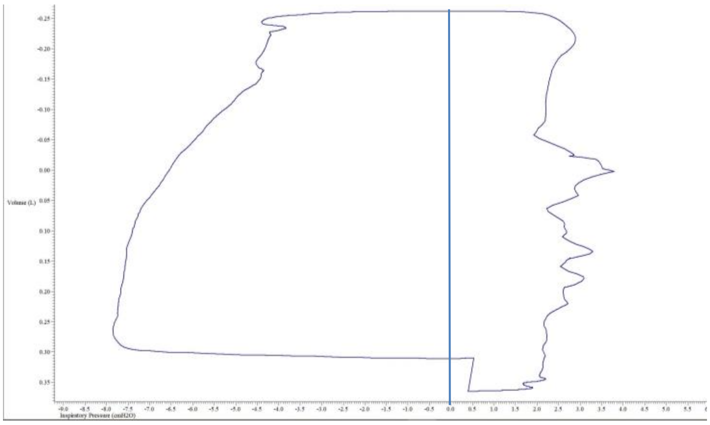

Example of the comparison of the calculation of the work of breathing performed on the raw signal of the portable spirometer (Panel A) and the calculation of the smoothed signal with a time constant of 0.13s in the Spike 2 software (Panel B).
