# Supplementary material for: Measurement validity of an electronic training device to assess breathing characteristics during inspiratory muscle training in patients with weaning difficulties
Source: PLoS One. 2021 Aug 26;16(8):e0255431. doi: 10.1371/journal.pone.0255431 (PMC8389486; doi:10.1371/journal.pone.0255431)
Supplement: S2 Fig — Comparison of tidal volume, mean inspiratory flow and peak inspiratory flow. Breaths that were recorded by the portable spirometer (n = 937) were compared to breaths that were recorded by portable spirometer but missed by the inspiratory training device (n = 65) and compared to all the breaths recorded by the inspiratory training device (n = 937). Red line indicates the median. EITD: electronic inspiratory training device. (PDF) [file pone.0255431.s002.pdf]

S2 Fig. Comparison between recorded breaths and breaths that were not detected by the inspiratory training device.

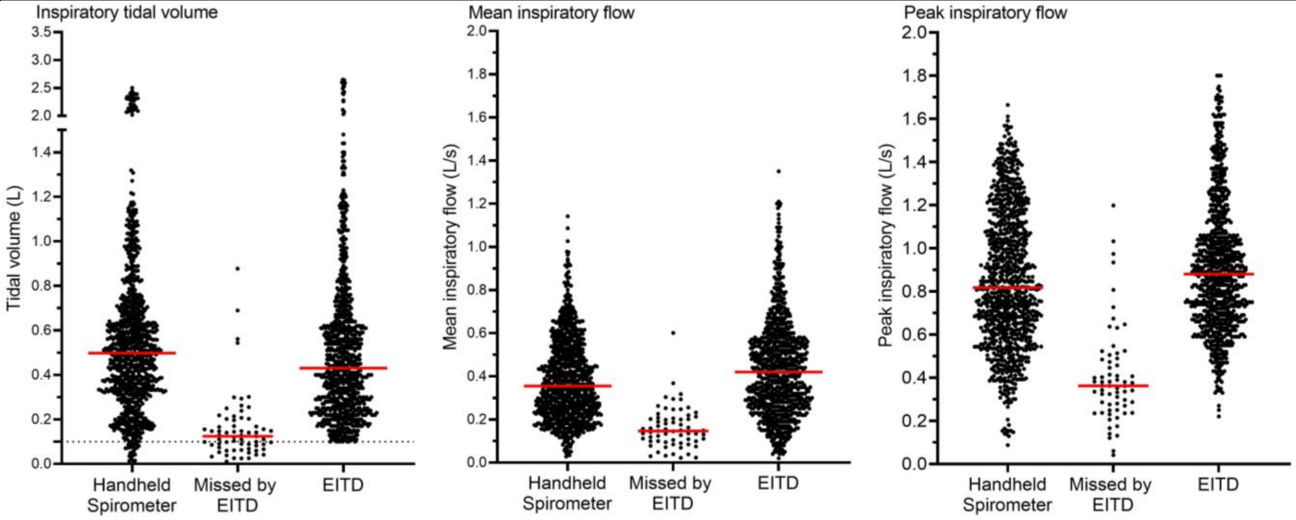

Comparison of tidal volume, mean inspiratory flow and peak inspiratory flow. Breaths that were recorded by the portable spirometer (n= 937) were compared to breaths that were recorded by portable spirometer but missed by the inspiratory training device (n=65) and compared to all the breaths recorded by the inspiratory training device (n=937). Red line indicates the median. EITD: electronic inspiratory training device.
