# Supplementary material for: Measurement validity of an electronic training device to assess breathing characteristics during inspiratory muscle training in patients with weaning difficulties
Source: PLoS One. 2021 Aug 26;16(8):e0255431. doi: 10.1371/journal.pone.0255431 (PMC8389486; doi:10.1371/journal.pone.0255431)
Supplement: S1 File — (DOCX) [file pone.0255431.s003.docx]

# Logistic regression analysis of breathing characteristics associated with breaths not being detected by the inspiratory training device

| **Table S1: Possible predictors of the inspiratory loading device detecting breaths** | | | | | | | | | |
| --- | --- | --- | --- | --- | --- | --- | --- | --- | --- |
| **Variable** | | | **B** | **S.E.** | **Wald** | **df** | **p-value** | **Odds ratio** | **95% CI for odds ratio** |
| **Inspiratory tidal volume <0.1L** | | | 1.10 | 0.43 | 6.63 | 1 | 0.01* | 3.0 | 1.30 to 6.91 |
| **Peak inspiratory Flow (dL/s)** | | | 0.41 | 0.12 | 10.89 | 1 | 0.001* | 1.51 | 1.18 to 1.92 |
| **Mean inspiratory flow (dL/s)** | | | 0.35 | 0.27 | 1.69 | 1 | 0.19 | 1.41 | 0.84 to 2.39 |
| **Peak inspiratory pressure (cmH_2_O)** | | | -0.03 | 0.06 | 0.25 | 1 | 0.62 | 0.97 | 0.87 to 1.09 |
| **Mean inspiratory pressure ( cmH_2_O)** | | | 0.03 | 0.11 | 0.07 | 1 | 0.79 | 1.03 | 0.84 to 1.27 |
| **Intercept** | | | 0.37 | 0.61 | 0.37 | 1 | 0.54 | 1.45 |  |
| A logistic regression was used to identify breathing characteristics that predict the breaths that are not detected by the electronic inspiratory loading device. Inspiratory tidal volume was entered as a dichotomous variable in which tidal volume ≥0.1L was marked as 0 and tidal volume <0.1L was marked as 1. B: logistic regression model coefficients, S.E.: coefficient’s standard error, df: degrees of freedom, 95% CI: 95% confidence intervals, *p<0.05 | | | | | | | | | |
|  |  |  | | | | | | | |

| **Table S2: Elimination of breathing characteristics that do not have an effect on the detecting of breaths** | | | | | | | | | |
| --- | --- | --- | --- | --- | --- | --- | --- | --- | --- |
| **Variable** | | | **B** | **S.E.** | **Wald** | **df** | **p-value** | **Odds ratio** | **95% CI for odds ratio** |
| **Inspiratory tidal volume <0.1L** | | | 1.20 | 0.40 | 9.09 | 1 | 0.003* | 3.32 | 1.52 to 7.25 |
| **Peak inspiratory Flow (dL/s)** | | | 0.54 | 0.08 | 42.91 | 1 | <0.001* | 1.71 | 1.46 to 2.00 |
| **Intercept** | | | 0.40 | 0.47 | 0.71 | 1 | 0.40 | 1.49 |  |
| A backward stepwise logistic regression was used to eliminate the breathing characteristics that do not have a significant effect on the missing of breaths by the electronic inspiratory training device. Inspiratory tidal volume was entered as a dichotomous variable in which tidal volume ≥0.1L was marked as 0 and tidal volume <0.1L was marked as 1. B: logistic regression model coefficients, S.E.: coefficient’s standard error, df: degrees of freedom, 95% CI: 95% confidence intervals, *p<0.05 | | | | | | | | | |
|  |  |  | | | | | | | |
