# Supplementary material for: Measurement validity of an electronic training device to assess breathing characteristics during inspiratory muscle training in patients with weaning difficulties
Source: PLoS One. 2021 Aug 26;16(8):e0255431. doi: 10.1371/journal.pone.0255431 (PMC8389486; doi:10.1371/journal.pone.0255431)
Supplement: Attachment — Submitted filename: Response to Reviewers21_07_07.docx [file pone.0255431.s004.docx]

**Response**

**Journal Requirements:**

1. Please ensure that your manuscript meets PLOS ONE's style requirements, including those for file naming. The PLOS ONE style templates can be found at
   <https://journals.plos.org/plosone/s/file?id=wjVg/PLOSOne_formatting_sample_main_body.pdf> and
   <https://journals.plos.org/plosone/s/file?id=ba62/PLOSOne_formatting_sample_title_authors_affiliations.pdf>

**Answer:** Changes have been made across the whole manuscript to be in accordance to the style requirements of PLOS ONE:

- Titles of all authors have been removed (Lines 5 and 6)
- Annotation of contact information of the corresponding author has been changed (lines 16-19)
- Author contribution statement has been removed (lines 21-22)
- Subheadings in the abstract have been removed (Lines 24, 33, 39 and 45).
- Italic text formatting has been removed on line 246, 249 and 250 to be in accordance with the style requirements.
- The headings “Acknowledgements” and “References” have been changed from a level 2 heading to a level 1 heading according to the style requirements (Line 263 and 269)
- The line spacing in the manuscript has been changed from a 1.5 spacing to a double spacing.

2. Please review your reference list to ensure that it is complete and correct. If you have cited papers that have been retracted, please include the rationale for doing so in the manuscript text, or remove these references and replace them with relevant current references. Any changes to the reference list should be mentioned in the rebuttal letter that accompanies your revised manuscript. If you need to cite a retracted article, indicate the article’s retracted status in the References list and also include a citation and full reference for the retraction notice.

**Answer:** The reference list has been checked and is complete and correct:

Reference 6: Guimarães BDS, de Souza LC, Cordeiro HF, Regis TL, Leite CA, Puga FP, et al. Inspiratory Muscle Training With an Electronic Resistive Loading Device Improves Prolonged Weaning Outcomes in a Randomized Controlled Trial. Critical care medicine. 2020;Publish Ahead of Print. doi: 10.1097/CCM.0000000000004787 is a reference referring to an article published ahead of print and has now been updated (line 290-293)

3. Please include in your Methods details of the consent procedure. Please include information on whether the IRB specifically approved the consent procedure, and whether it was in line with national regulations.

**Answer:** Information on the consent procedure has been added to the manuscript on lines 79 to 81.

4. Thank you for stating the following in the Competing Interests section:
   [I have read the journal's policy and the authors of this manuscript have the following competing interests: the POWERbreathe KH2 devices, used in this study were provided by HAB - POWERbreathe International Ltd., (Warwickshire, England) for study purposes at no additional cost.].

   Please confirm that this does not alter your adherence to all PLOS ONE policies on sharing data and materials, by including the following statement: "This does not alter our adherence to  PLOS ONE policies on sharing data and materials.” (as detailed online in our guide for authors [http://journals.plos.org/plosone/s/competing-interests](about:blank)).  If there are restrictions on sharing of data and/or materials, please state these. Please note that we cannot proceed with consideration of your article until this information has been declared.

   Please include your updated Competing Interests statement in your cover letter; we will change the online submission form on your behalf.

   Please know it is PLOS ONE policy for corresponding authors to declare, on behalf of all authors, all potential competing interests for the purposes of transparency. PLOS defines a competing interest as anything that interferes with, or could reasonably be perceived as interfering with, the full and objective presentation, peer review, editorial decision-making, or publication of research or non-research articles submitted to one of the journals. Competing interests can be financial or non-financial, professional, or personal. Competing interests can arise in relationship to an organization or another person. Please follow this link to our website for more details on competing interests: [http://journals.plos.org/plosone/s/competing-interests](about:blank)

**Answer:** Hereby, I confirm that the competing interest addressed does not alter our adherence to PLOS ONE policies on sharing data and materials. The updated Competing Interests statement has been added to the cover letter.

5. In your Data Availability statement, you have not specified where the minimal data set underlying the results described in your manuscript can be found. PLOS defines a study's minimal data set as the underlying data used to reach the conclusions drawn in the manuscript and any additional data required to replicate the reported study findings in their entirety. All PLOS journals require that the minimal data set be made fully available. For more information about our data policy, please see <http://journals.plos.org/plosone/s/data-availability>.

   Upon re-submitting your revised manuscript, please upload your study’s minimal underlying data set as either Supporting Information files or to a stable, public repository and include the relevant URLs, DOIs, or accession numbers within your revised cover letter. For a list of acceptable repositories, please see <http://journals.plos.org/plosone/s/data-availability#loc-recommended-repositories>. Any potentially identifying patient information must be fully anonymized.

   Important: If there are ethical or legal restrictions to sharing your data publicly, please explain these restrictions in detail. Please see our guidelines for more information on what we consider unacceptable restrictions to publicly sharing data: <http://journals.plos.org/plosone/s/data-availability#loc-unacceptable-data-access-restrictions>. Note that it is not acceptable for the authors to be the sole named individuals responsible for ensuring data access.

   We will update your Data Availability statement to reflect the information you provide in your cover letter.

**Answer:** The data availability statement has been updated in the cover letter including the relevant DOI to access the data.

6. We note that Figure 1 B includes an image of a patient and a nurse.
   As per the PLOS ONE policy ([http://journals.plos.org/plosone/s/submission-guidelines#loc-human-subjects-research](about:blank#loc-human-subjects-research)) on papers that include identifying, or potentially identifying, information, the individual(s) or parent(s)/guardian(s) must be informed of the terms of the PLOS open-access (CC-BY) license and provide specific permission for publication of these details under the terms of this license. Please download the [Consent Form for Publication in a PLOS Journal](about:blank) ([http://journals.plos.org/plosone/s/file?id=8ce6/plos-consent-form-english.pdf](about:blank)). The signed consent form should not be submitted with the manuscript, but should be securely filed in the individual's case notes. Please amend the methods section and ethics statement of the manuscript to explicitly state that the patient/participant has provided consent for publication: “The individual in this manuscript has given written informed consent (as outlined in PLOS consent form) to publish these case details”.

   If you are unable to obtain consent from the subject of the photograph, you will need to remove the figure and any other textual identifying information or case descriptions for this individual.

**Answer:** Statement on the consent for publication of the patient in figure 1B has been added to the method section (line 81-82). The patient included in the image has been informed of the terms of the PLOS open-access license and signed the consent form for publication in a PLOS Journal. The therapist included in the figure is the first author of the current study (Marine Van Hollebeke) and has signed the consent form for publication in a PLOS Journal.

**Response to reviewers**

Reviewer #1: 28-Jun-2021

PLOS ONE
PONE-D-21-15405: Measurement validity of an electronic training device to assess breathing characteristics during inspiratory muscle training in weaning failure patients
This is a manuscript that does not raise a clinical question. It was designed to compare the measurements of selected respiratory parameters obtained by a new electronic device (Powerbreathe KH2, POWERbreathe International Ltd, UK) with the ones obtained more traditionally, using a portable spirometer (Pocket-Spiro USB/BT 100, M.E.C, Belgium). The authors aimed to validate the measures of the new device. The question brought about by the study is relevant considering that it checks if a device with a potentially wide application generates reliable numbers.
The study succeeds in its general purpose, but below are some comments to clarify some points posed in the manuscript.

1. In the title. Weaning failure is more an event than a stable situation. It would be convenient to replace “weaning failure” by “difficult weaning”. In this sense, this change would apply to the whole manuscript.

**Answer:** Thank you for taking the time to read the manuscript and to provide us with valuable feedback. I agree that weaning failure is more an event than a stable condition and that it is appropriate to indicate that we included patients with weaning difficulties. However, I would avoid using the exact term “difficult weaning” as this term has also been used as one of the possible classification groups in the WIND study (1). As we used this classification system and included both patients from the difficult weaning and the prolonged weaning group, I will avoid using “difficult weaning” but instead using the term “weaning difficulties”. Changes have been made in the title (line 1) and across the manuscript (lines: 26, 32, 34, 48, 55, 65, 70, 75, 201, 203, 218, 219, 235, 256) to change ‘weaning failure’ to indicate that patients have ‘weaning difficulties’.

(1) Beduneau G, Pham T, Schortgen F, Piquilloud L, Zogheib E, Jonas M, et al. Epidemiology of Weaning Outcome according to a New Definition. The WIND Study. *Am J Respir Crit Care Med* (2017) 195(6):772-83. Epub 2016/09/15. doi: 10.1164/rccm.201602-0320OC. PubMed PMID: 27626706.
2. In the abstract. Both devices (the EITD and the spirometer) could have their model and manufacturer mentioned here.

**Answer:** On line 36 to 38, the model and manufacturer of the training device and the portable spirometer have been added to the abstract.

3. Line 135. Replace the word confident por confidence

**Answer:** Thank you for noticing this error. Changes have been made on line 131

4. Line 137. Replace the word “patients effects” with “patients' effects”.

**Answer:** Thank you for noticing this error. Changes have been made on line 133

5. Table 1. Please replace “VC” by “VT”. Abbreviations “VT” and “PImax” need to be explicated in the table legend.

**Answer:** Thank you for this comment and helping to improve the quality of the manuscript. The abbreviations are now explicated in the legend of Table 1. By changing “VC” to “VT”, I assume the purpose would be to indicate that this value represents the tidal volume. I can see that this value might be mistaken for the tidal volume due to the low value (0.9L). However, this value actually represents the forced vital capacity. To clarify this in the table I have changed the abbreviation from “VC” to “FVC”. In addition, I noticed that we have mentioned the abbreviation BMI in the legend but do not provide the value for BMI in the table. Instead we chose to provide body mass and height separately. Therefore, I deleted BMI out of the legend of Table 1.

Reviewer #2:

This is an interesting study which concludes that an electronic inspiratory training device (EITD; POWERbreathe KH2) provides valid measurements of breathing characteristics (inspiratory muscle work and peak power) when compared to the gold standard device (Pocket-spiro) during inspiratory muscle training in weaning failure patients. The important clinical implications of this study are clear, and relate to the ability of the EITD to provide breathing characteristics during training in real-time, allowing for therapists to optimise the training stimulus during every training session, and helping motivate the patient via visual feedback.

1. The manuscript is well written and the analyses were well conducted. My main query with this study relates to the disparity of the sample size within this manuscript and the abstract presented by the authors at the European Respiratory Society (ERS) congress 2020 (included in the European Respiratory Journal proceedings ) entitled “Measurement validity of an electronic inspiratory loading device during inspiratory muscle training in weaning failure patients” [1]. Within the congress abstract there are 30 participants included, with 2594 breaths from 64 training sessions analysed compared to 13 participants and 1002 breaths from 27 training sessions analysed in the current manuscript. The findings of both versions are similar, with an even higher ICC and lower relative bias (% difference) between the devices within the ERS abstract for most variables. However, mean inspiratory resistance during training was much lower in the ERS abstract (24% of maximal inspiratory pressure; PImax) compared to this manuscript (46% PImax). Please could you comment on the reason behind the lower sample size and higher training intensity within this manuscript compared to previously reported results [1]. Were training sessions at a lower inspiratory resistance (i.e. lower % PImax) excluded from the analysis within this manuscript and could this have affected the results in any way?
   1. Van Hollebeke, M., Poddighe, D., Clerckx, B., Hermans, G., Gosselink, R., & Langer, D. (2020). Measurement validity of an electronic inspiratory loading device during inspiratory muscle training in weaning failure patients.

**Answer:** Thank you for taking the time to provide us with feedback on the manuscript and for noticing the disparity of the sample size between the manuscript and the abstract previously presented at the ERS congress (1). The patients included in the present study were recruited from an ongoing randomized controlled trial (2). In this RCT, patients were randomized in an intervention group (high-intensity IMT) or a control group (sham low-intensity IMT). In the intervention group, patients performed IMT with a mean training load of 30-50%PImax and in the control group, patients performed IMT with a training load of maximal 10% PImax. In the abstract presented on the ERS congress, we included data from patients from both the intervention group (n=13) and the control group (n=17).

However, due to valuable feedback during the ERS congress and in consultation with the study team we decided in a later stage to focus the message of the manuscript on the validity of the data on breathing characteristics during IMT of patients randomized in the intervention group only.

In a clinical setting IMT will be currently performed similarly to the training protocol used in the intervention group (high-intensity IMT).

Our main aim with the current validity study was to identify whether the IMT device provides valid feedback during IMT to allow the clinician to optimize the training intensity during every training session. Therefore, we focused only on the intervention group, as this is more clinically relevant. Additionally by including both groups, the training intensity is bimodally distributed, as the training intensity is lower than 10% PImax in the control group and between 30 to 50% in the intervention group. This leads to a mean training load of approximately 24%PImax.

By excluding, the patients randomized in the control group for the present study we aimed improved the clinical message of the validity study.

(1) Van Hollebeke, M., Poddighe, D., Clerckx, B., Hermans, G., Gosselink, R., & Langer, D. (2020). Measurement validity of an electronic inspiratory loading device during inspiratory muscle training in weaning failure patients.
(2) Hoffman M, Van Hollebeke M, Clerckx B, Muller J, Louvaris Z, Gosselink R, et al. Can inspiratory muscle training improve weaning outcomes in difficult to wean patients? A protocol for a randomised controlled trial (IMweanT study). BMJ Open (2018) 8(6):e021091. Epub 2018/07/02. doi: 10.1136/bmjopen-2017-021091. PubMed PMID: 29961023; PubMed Central PMCID: PMCPMC6042566.
2. Specific comments:
   Table S1. – For the peak inspiratory pressure logistic regression model coefficient, I think there is a typo - please clarify the value.

**Answer:** Thank you for noticing this error. Changes have been made in table S1. The error in the logistic regression model coefficient of the peak inspiratory pressure has been changed from -0..3 to -0.03.
